# Identification of Specific Cervical Cancer Subtypes and Prognostic Gene Sets in Tumor and Nontumor Tissues Based on GSVA Analysis

**DOI:** 10.1155/2022/6951885

**Published:** 2022-10-15

**Authors:** Zihang Zhong, Yuanyuan Wang, Jian Yin, Senmiao Ni, Wen Liu, Rui Geng, Jinhui Liu, Jianling Bai, Hao Yu

**Affiliations:** ^1^Department of Biostatistics, School of Public Health, Nanjing Medical University, 101 Longmian Avenue, Jiangning District, Nanjing 211166, China; ^2^Department of Gynecology, The First Affiliated Hospital of Nanjing Medical University, Nanjing, 210029 Jiangsu, China

## Abstract

**Background:**

Cervical cancer is the fourth common cancer among women. Its prognosis needs our more attention. Our purpose was to identity new prognostic gene sets to help other researchers develop more effective treatment for cervical cancer patients and improve the prognosis of patients.

**Methods:**

We used gene set variation analysis (GSVA) to calculate the enrichment scores of gene sets and identified three subtypes of cervical cancer through the Cox regression model, k-means clustering algorithm, and nonnegative matrix factorization method (NMF). Chi-square test was utilized to test whether a certain clinical characteristic is different among divided subtypes. We further screened the prognostic gene sets using differential analysis, univariate Cox regression analysis, and least absolute shrinkage and selection operator (LASSO) regression. Gene Ontology (GO) and Kyoto Encyclopedia of Genes and Genomes (KEGG) enrichment analyses were used to analyze which pathways and function the genes from screened gene sets enriched. Search Tool for the Retrieval of Interacting Genes (STRING) was used to draw the protein-protein interaction network, and Cytoscape was used to visualize the hub genes of protein-protein interaction network.

**Results:**

We identified three novel subtypes of cervical cancer in The Cancer Genome Atlas (TCGA) samples and validated in Gene Expression Omnibus (GEO) samples. There were significant variations between the three subtypes in histological type, T stage, M stage, and N stage. T_GSE36888_UNTREATED_VS_IL2_TREATED_STAT5_AB_KNOCKIN_TCELL_2H_UP and N_HALLMARK_ANGIOGENESIS were screened prognostic gene sets. The prognostic model was as follows: riskScore = T_GSE36888_UNTREATED_VS_IL2_TREATED_STAT5_AB_KNOCKIN_TCELL_2H_UP^∗^ 2.617 + N_HALLMARK_ANGIOGENESIS^∗^ 4.860. Survival analysis presented that in these two gene sets, high enrichment scores were all significantly related to worse overall survival. The hub genes from T gene set included CXCL1, CXCL2, CXCL8, ALDOA, TALDO1, LDHA, CCL4, FCAR, FCER1G, SAMSN1, LILRB1, SH3PXD2B, PPM1N, PKM, and FKBP4. As for N gene sets, the hub genes included ITGAV, PTK2, SPP1, THBD, and APOH.

**Conclusions:**

Three novel subtypes and two prognostic gene sets were identified. 15 hub genes for T gene set and 5 hub genes for N gene set were discovered. Based on these findings, we can develop more and more effective treatments for cervical cancer patients. Based on the gene enriched pathways, we can development specific drugs targeting the pathways.

## 1. Introduction

Globally, cervical cancer recently has been ranked as the fourth most common cancer against women, with estimated 570,000 cases and 311,000 deaths in 2018 [1]. Cervical cancer is a serious health-threatening female disease, especially for those ages from 20 to 39, which remains to be the second leading cause to their mortality resulted from cancer [2]. In China, the incidence and mortality of cervical cancer in young women continue rapidly increasing [3]. Early cervical cancer can be effectively treated with classic surgery, while the outcome for metastatic cervical cancer is poor, for which the 5-year survival rate is only 16.5% and the median survival time is only 8-13 months [4, 5]. Therefore, the efficient approaches for the stratification and biomarker screening of cervical cancer patients are urgently needed.

Fortunately, as it has been observed in many studies in recent years, genetic factors can be closely related with the susceptibility and tumorigenesis of cervical cancer [6, 7], and increasing researches have been focused on the correlation of cervical cancer prognosis and survival with potential molecule indicators. Moreover, further discovery and understanding of those factors can help to guide treatment decision and determine the personalized therapeutic targets, thus improving the treatment effects for longer lifespan of patients.

The gene set variation analysis (GSVA) is a nonparametric and unsupervised gene set enrichment method, assaying the variation of gene set enrichment over sample population, thus condensing gene expression profiles into gene set or pathway summary [8]. Using GSVA can integrate the prognostic genes into a complex or pathways for advanced analysis, which can be more convenient for following statistics calculation and pathogenesis inferences [9]. GSVA method has been utilized in survival-associated gene mechanism researches for breast cancer [10], colon cancer [11], bladder cancer [12], and so on.

In this study, we selected the sequencing data of cervical cancer patients from several cohort in The Cancer Genome Atlas (TCGA) and Gene Expression Omnibus (GEO) databases for prognostic analysis. The cervical cancer was automatically classified into 3 subtypes, and they revealed different features in some clinical traits like tumor T, N, and M stages. Moreover, the data were screened to identify the prognostic relevant pathways and gene sets in tumor and nontumor tissues by differential analysis, univariate Cox regression analysis, and the least absolute shrinkage and selection operator (LASSO) regression. As a result, one tumor (T gene set) and one nontumor (N gene set) gene sets were found, and high enrichment scores of them were all strongly associated with poor overall survival. Functional enrichment analysis and protein-protein network analysis for the hub genes were conducted to investigate the possible regulatory mechanisms. The results indicated that the genes in T gene set were connected with immune activity and metabolic process, while the genes in N gene set were related to angiogenesis and protein regulation. Our findings may be helpful for uncovering the biomarkers of effective prognosis and potential therapeutic targets for precise treatment of cervical cancer patients.

## 2. Methods

### 2.1. Data Source

The transcriptome data from TCGA-CESC (HT-Sequence-FPKM) were downloaded from TCGA website (http://portal.gdc.cancer.gov). The clinical data of cervical cancer in TCGA database [13] were downloaded from UCSC Xena (http://xena.ucsc.edu/). The cervical cancer datasets GSE44001 were downloaded from Gene Expression Omnibus (GEO) database (https://www.ncbi.nlm.nih.gov/geo/) [14]. The data of gene expression in TCGA cohort contained 3 adjacent nontumor samples and 306 tumor samples, and GEO cohort contained 300 tumor samples. The clinical data contained 308 samples in TCGA cohort after removing duplicates. The following criteria were used for exclusion: (1) the histological diagnosis was not standard and (2) the specimens did not have complete clinical data available. 4922 gene sets were downloaded from MSigDB [15] for GSVA analysis.

### 2.2. Gene Set Variation Analysis (GSVA)

GSVA can detect the slight pathway activity changes within large number of gene sets [8]. It transforms the expression matrix of genes in different samples into the enrichment scores of gene sets to evaluate the enrichment of gene sets [9]. In this study, we used GSVA package in R to do gene set variation analysis, and a gene set c7.immunesigdb_HALLMARK related to immunity was used in GSVA. c7.immunesigdb_HALLMARK contains 4922 gene sets and the names of genes which they contained.

### 2.3. The Identification of Cancer Subtypes and Differential Analysis of Gene Sets

The CancerSubtypes package [16] in R was used on the enrichment scores of tumor gene sets and combined survival data from TCGA database and validated on GEO data (GSE44001) to identify cancer subtypes.

First of all, we used the Cox regression model to do the biological feature selection through CancerSubtypes package in R by “FSbyCox” function. Then, NbClust package was utilized to discover the optimal number of clusters and visualize it. The nonnegative matrix factorization (NMF) was used to reduce the dimensions of complex data and provide a powerful assistance for clustering [17]. Based on the optimal number of clusters we found, we used k-means clustering algorithm to conduct the clustering analysis and draw a cluster diagram through NMF [18] and factoextra package. Survival analysis was applied to discover the difference among the divided subtypes, and the Kaplan-Meier plot was plotted at the same time.

The cluster heatmap was drawn on combined clinicopathological data and enrichment scores of gene sets through pheatmap package. The cluster heatmap presented the correlation between clinical characteristics and divided subtypes. Chi-square test was utilized to test whether a certain clinical characteristic is different among divided subtypes.

The differential analysis of gene sets was performed with limma package and visualized through VennDiagram package. Then, based on the number of differential gene sets we found, the heatmap of represented gene sets in divided subtypes was drawn through pheatmap package in R.

### 2.4. Screening of Prognosis-Related Gene Sets

First of all, the univariable Cox regression analysis and log-rank test were exerted to screen potentially prognosis-related gene sets (*p* < 0.05) within TCGA cohort. Then, combined with survival data in GEO cohort, we further explored central parts of the screened prognosis-related gene sets using LASSO regression [19] through Glmnet package. The screened prognostic gene sets were employed to construct the prognostic model. In the prognostic model, risk score was the dependent variable, and the enrichment scores of the screened prognostic gene sets were the independent variables. The risk score was calculated according to the following formula: ∑_*i*=1_^*n*^Coef_*i*_∗*x*_*i*_. Coef is the coefficient of the Cox regression analysis.

For those prognosis-related gene sets in the model, we employed survival analysis to figure out their association with overall survival.

### 2.5. GO and KEGG Enrichment Analyses

Functional enrichment analysis was performed to investigate the possible mechanism of the genes extracted from the screened gene sets and which function or pathways the genes participate in. Gene Ontology (GO) enrichment analysis [20] was used to identify biological functions of the genes, including biological processes (BP), cellular components (CC), and molecular functions (MF). The Kyoto Encyclopedia of Genes and Genomes (KEGG) enrichment analysis [21] was used to explore the enriched pathways. Therefore, “enrichGO” and “enrichKEGG” were the functions from R package “clusterProfiler” for GO and KEGG analyses, respectively. The results were then visualized using packages “pheatmap” and “ggplot2.”

### 2.6. Construct Protein-Protein Interaction Network and Identify the Hub Genes

We applied the Search Tool for the Retrieval of Interacting Genes (STRING) database to help us construct the protein-protein interaction (PPI) networks for genes from the specific gene sets in obtained risk model [22]. Genes for each gene set were put into STRING to visualize PPI networks, and the results were further imported into Cytoscape software for the search of significant module [23]. We used the Molecular Complex Detection (MCODE) to discover the modules and determine the hub genes in each cluster. The hub genes were marked in red.

### 2.7. Statistical Analysis

All the analyses were conducted using R (version 4.1.2). The classified data were summarized in the form of counts (percentage). The results were considered to be significant when *p* is lower 0.05.

## 3. Results

### 3.1. The Activity Changes of Gene Sets in Cervical Cancer and Adjacent Normal Samples

**Figure 1:**
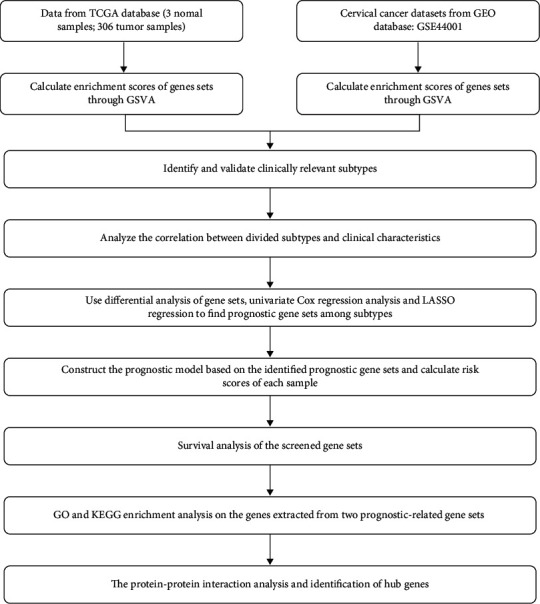
Flowchart of this study.

Figure 1 is the flowchart of our study.

**Figure 2:**
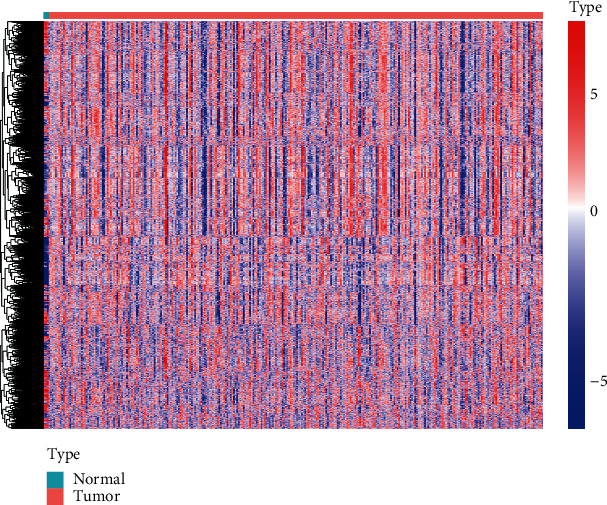
The heatmap of enrichment score of 4922 gene sets in cervical cancer and adjacent normal samples.

We first downloaded the transcriptome data (N = 3 and T = 306) of cervical cancer from TCGA website (http://portal.gdc.cancer.gov) and then got 4299 gene set expression data. The enrichment scores of 4299 immune-related gene sets were calculated by GSVA and were shown in the following heatmap (Figure 2). In normal samples, most part of the gene sets were upregulated and some part of them were downregulated. As shown in Figure 2, these gene sets can be classified into different categories with high probability.

### 3.2. The Identification of Clinically Relevant Subtypes of Cervical Cancer Patients

**Figure 3:**
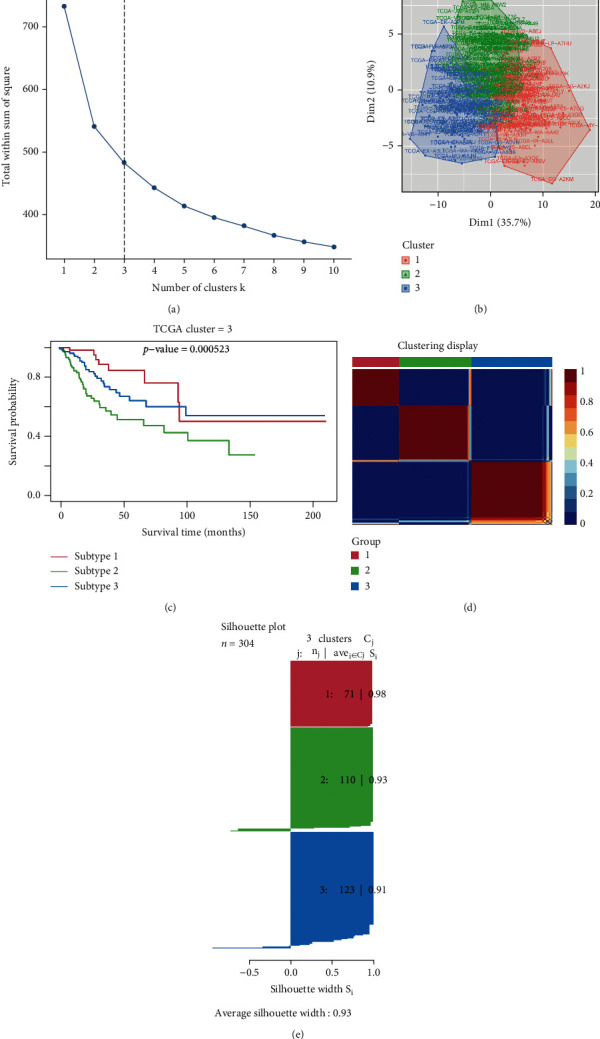
Identification of clinically relevant subtypes of cervical cancer in TCGA database. (a) The optimal number of clusters (*k*). (b) The cluster plot of 3 subtypes. (c) Kaplan-Meier plot of the three clusters. (d) The correlation heatmap of 3 subtypes. (e) Silhouette plot of three subtypes.

Therefore, we paid attention to classify cervical cancer patients into different subtypes. There is one patient who was removed for lack of prognostic information in clinical data (307 remained). Through “avereps” function in limma package, a microarray data object was condensed, and there were 304 gene set expression samples left. Based on the enrichment scores calculated by GSVA and the prognostic information, we used the Cox regression to do the feature selection through CancerSubtypes package. We got the optimal number of clusters (*k* = 3), and the result is exhibited in Figure 3(a). Then, Figure 3(b) visualizes the three divided subtypes. As presented in Figure 3(c), cervical cancer patients with subtypes 1 and 3 have better overall survival than patients with subtype 2. As exhibited in Figure 3(d), one subtype had little relationship with other subtypes, illustrating a great performance for clustering cervical cancer patients into three subtypes. The average silhouette width value was 0.93 in silhouette plot (Figure 3(e)), demonstrating that clustering cervical cancer patients into three subtypes can be more accurate. Subsequently, we used data from GEO database to validate the three subtype classifications (Figure S1), and the results showed that the classification was appropriate. Comparing Figure 3(c) with Figure S1C, we can find that the subtype 1,3,2 which was classified using data from TCGA database corresponds to subtype 2,1,3 which was classified using data from GEO database, respectively.

### 3.3. Correlation between Clinical Characteristics and Cervical Cancer Subtypes

**Figure 4:**
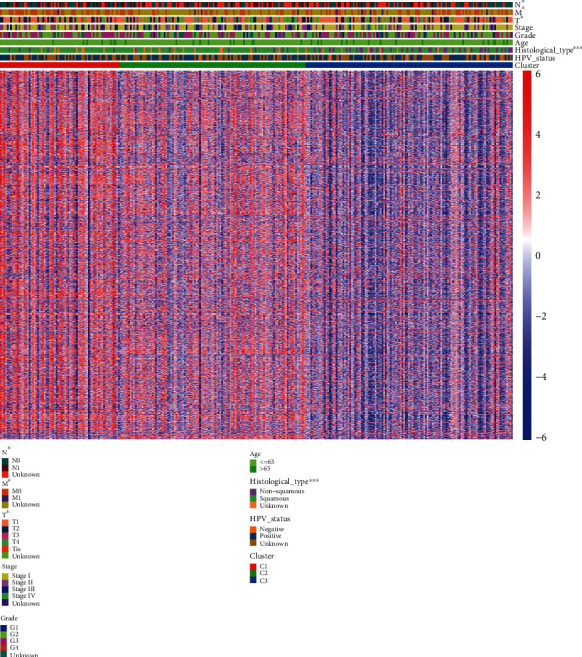
The correlation between enrichment scores of gene sets and clinical characteristics in TCGA cohort. ^∗∗∗^*p* < 0.001, ^∗∗^*p* < 0.01, and ^∗^*p* < 0.05.

Then, we further investigated the correlation between clinical characteristics and the three divided cervical cancer subtypes. The clinicopathological features of three cervical cancer subtypes are presented in Figure 4 and Table 1.

The heatmap presented the distribution of clinical characteristics of each sample and the enrichment scores of gene sets within each sample (Figure 4). As shown in Figure 4, the enrichment scores of gene sets in subtype 1 were higher than other subtypes, suggesting that gene sets are upregulated in subtype 1. These findings showed that there were significant variations between the three subtypes in histological type, T stage, M stage, and N stage (Figure 4 and Table 1). Patients in subtypes 1, 2, and 3 all often have lower T, M, and N stages, although the T, N, and M stages of a lot of samples were unknown. Patients in subtypes 1 and 2 are almost cervical squamous cell carcinoma.

### 3.4. The Differential Analysis of Gene Sets within Three Divided Subtypes

**Figure 5:**
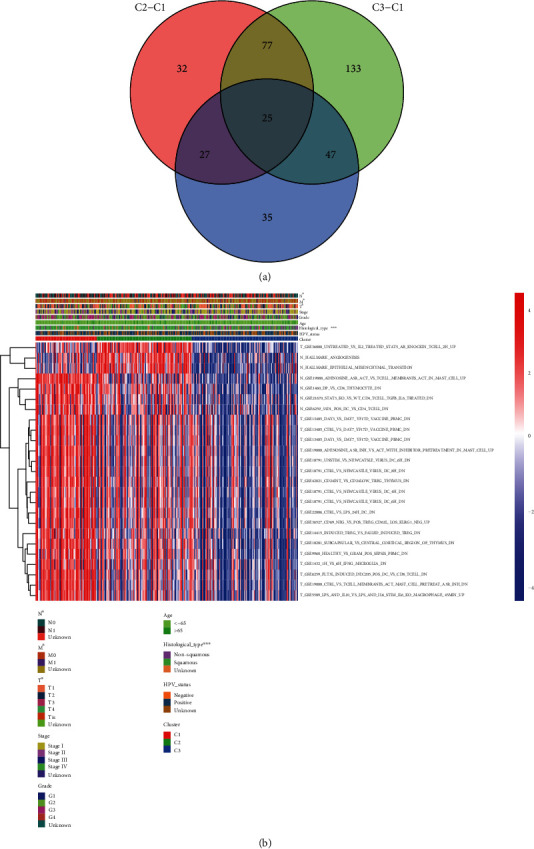
The differential analysis of gene sets. (a) Venn diagram of the number of differential gene sets between three subtypes. (b) Heatmap of represented gene sets in three subtypes.

Then, to find out the number of the differential gene sets among divided three subtypes, we calculated the discrepancy enrichment score and overlapped them (adjusted *p* value < 0.05) (Figure 5(a)). Finally, 25 differential gene sets were found to be representative, and they were distinct among the three subtypes. Based on the 25 differential gene sets we found, we draw the heatmap of the 25 differential gene sets and clinical characteristics (Figure 5(b)).

As displayed in Figure 5(b), subtype 1 had higher enrichment scores of 4 gene sets in nontumor samples and 18 gene sets in tumor samples than that in other subtypes, such as N_GSE1460_DP_VS_CD4_THYMOCYTE_DN, N_GSE21670_STAT3_KO_VS_WT_CD4_TCELL_TGFB_IL6_TREATED_DN, N_GSE6259_33D1_POS_DC_VS_CD4_TCELL_DN, T_GSE13485_DAY3_VS_DAY7_YF17D_VACCINE_PBMC_DN, T_GSE13485_DAY1_VS_DAY7_YF17D_VACCINE_PBMC_DN, and T_GSE19888_ADENOSINE_A3R_INH_VS_ACT_WITH_INHIBITOR_PRETREATMENT_IN_MAST_CELL_UP. Subtype 2 had higher enrichment scores of 2 gene sets in nontumor samples and 1 gene set in tumor samples, including T_GSE36888_UNTREATED_VS_IL2_TREATED_STAT5_AB_KNOCKIN_TCELL_2H_UP, N_HALLMARK_ANGIOGENESIS, and N_HALLMARK_EPITHELIAL_MESENCHYMAL_TRANSITION. Contrary to the two subtypes, subtype 3 had low enrichment scores in the 25 gene sets.

### 3.5. The Exploration of Prognostic Gene Sets

We used the univariate Cox regression analysis to screen potentially prognostic gene sets in TCGA cohort based on the expression of intersection gene sets from the results of differential analysis of gene sets. Ultimately, three gene sets were screened, and they were representative gene sets (*p* < 0.05), including N_HALLMARK_ANGIOGENESIS, N_HALLMARK_EPITHELIAL_MESENCHYMAL_TRANSITION, and T_GSE36888_UNTREATED_VS_IL2_TREATED_STAT5_AB_KNOCKIN_TCELL_2H_UP (Table S1). In Table S1, a logarithmic transformation of hazard ratio was also conducted. The hazard ratio of the three gene sets was larger than 1, suggesting the three gene sets have high survival risk for cervical cancer patients. The high expression the three gene sets had, the more dangerous the patients were. Combined with the results above, these three gene sets were all enriched in subtype 2, suggesting that the bad prognosis of patients in subtype 2 may be related to these three gene sets.

**Figure 6:**
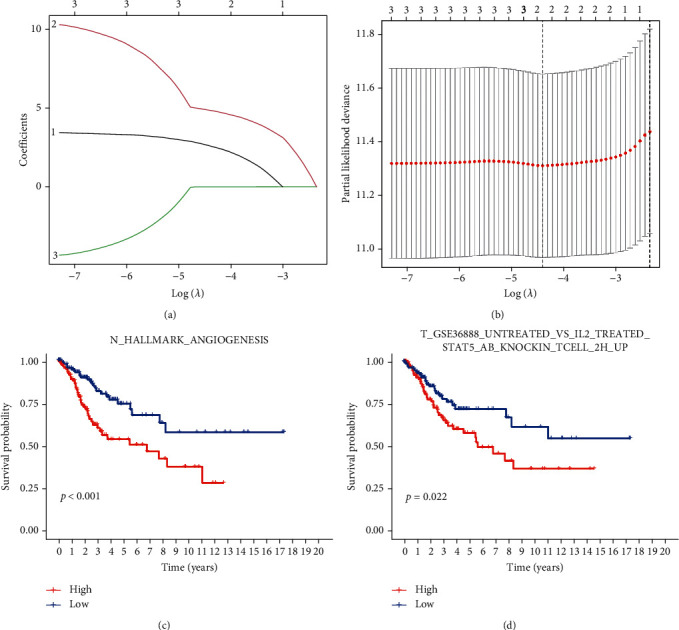
(a) Least absolute shrinkage and selection operator (LASSO) coefficient profiles of the gene sets. (b) Best penalization coefficient according to partial likelihood deviance. (c) K-M curves for N gene set and (d) T gene set.

Then, we used LASSO regression to find central parts of these three gene sets (Figures 6(a) and 6(b)), and two gene sets were identified with the corresponding risk coefficient (Table 2). One gene set was tumor gene set (T gene set: T_GSE36888_UNTREATED_VS_IL2_TREATED_STAT5_AB_KNOCKIN_TCELL_2H_UP), and the other was nontumor gene set (N gene set: N_HALLMARK_ANGIOGENESIS). Based on the enrichment scores of two prognostic gene sets and the risk coefficients (Table 2), we got the prognostic model: riskScore = T_GSE36888_UNTREATED_VS_IL2_TREATED_STAT5_AB_KNOCKIN_TCELL_2H_UP^∗^ 2.617 + N_HALLMARK_ANGIOGENESIS^∗^ 4.860.

Survival analysis for different groups classified by the median of enrichment scores was used to confirm the gene sets roles in prognosis, which are shown in Figures 6(c) and 6(d). The results indicated that in these two gene sets, high enrichment scores were both significantly related to worse overall survival time.

### 3.6. GO and KEGG Enrichment Analyses

Next, we executed GO and KEGG pathway enrichment analyses on the genes extracted from two prognostic-related gene sets for the further investigation of their mechanism. The two prognostic-related gene sets are screened from the differential gene sets among the three subtypes.

**Figure 7:**
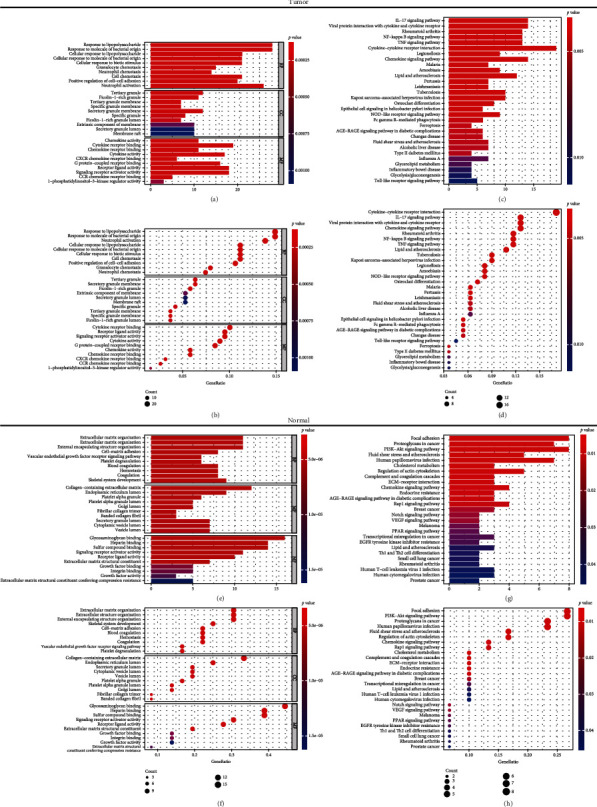
GO term and KEGG pathway analyses for genes from T and N gene sets. (a, b) Bar and bubble plot of GO term analysis and (c, d) of KEGG pathway analysis for genes from T gene sets. (e, f) Bar and bubble plot of GO term analysis and (g, h) of KEGG pathway analysis for genes from N gene sets.

The genes from T gene sets were shown involved in many processes in the analysis of GO biological process (Figures 7(a) and 7(b)). The top 3 enriched BP terms were response to lipopolysaccharide, response to molecule of bacterial origin, and neutrophil activation (Figures 7(a) and 7(b)). Cellular component enrichment analysis indicated that the tumor genes were closely associated with the CC terms including tertiary granule, secretory granule membrane, and ficolin-1-rich granule (Figures 7(a) and 7(b)). GO terms also revealed several molecular functions, including cytokine receptor binding, receptor ligand activity, and signaling receptor activator activity (Figures 7(a) and 7(b)). These results suggested that tumor tissue genes may lead to the changes in metabolism and immune. The KEGG pathway analysis demonstrated that immune-related pathways like cytokine-cytokine receptor interaction, IL-17 signaling pathway, and viral protein interaction with cytokine and cytokine receptor were the pathways most of tumor tissue genes enriched (Figures 7(c) and 7(d)), which indicated that they may impact on immune activity.

On the other hand, for nontumor genes, biological process indicated various terms such as extracellular matrix organization, extracellular structure organization, and external encapsulating structure organization related to genes from N gene sets (Figures 7(e) and 7(f)). Collagen-containing extracellular matrix, endoplasmic reticulum lumen, and secretory granule lumen were the top 3 CC terms for these genes (Figures 7(e) and 7(f)). The following MF terms shared close correlations with normal tissue genes: glycosaminoglycan binding, heparin binding, and sulfur compound binding (Figures 7(e) and 7(f)). In KEGG analysis, these genes were significantly enriched in focal adhesion, PI3K-Akt signaling pathway, and proteoglycans in cancer (Figures 7(g) and 7(h)).

### 3.7. Protein-Protein Interaction Analysis

**Figure 8:**
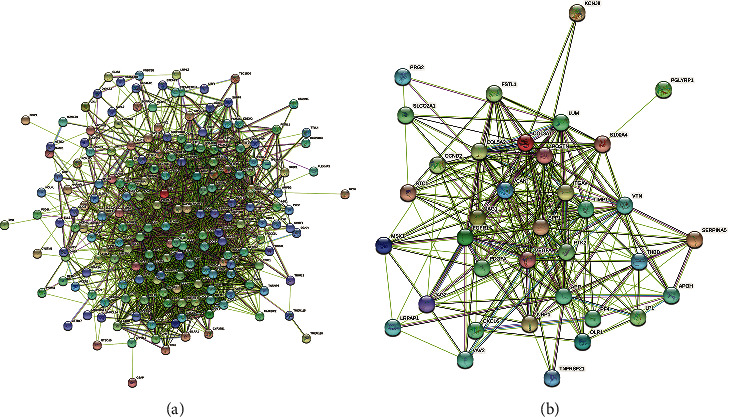
The protein-protein interaction network of (a) T gene sets and (b) N gene sets by STRING database.

**Figure 9:**
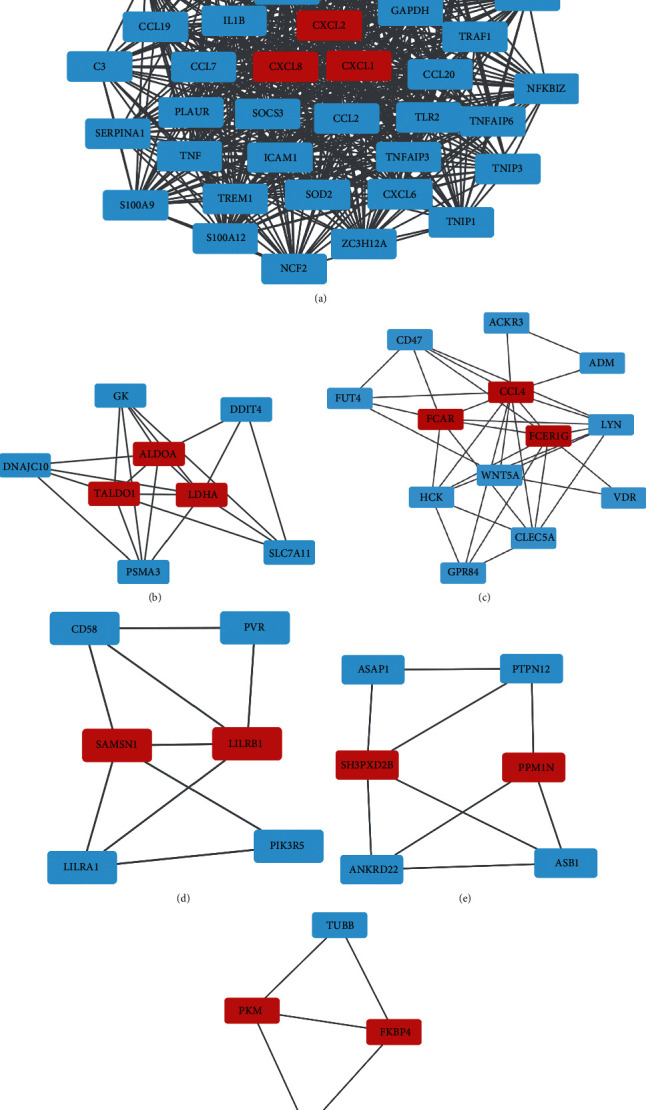
The (a–f) subnetworks 1-6 of protein-protein interaction network through MCODE of Cytoscape for T gene sets. The red represented the hub genes.

**Figure 10:**
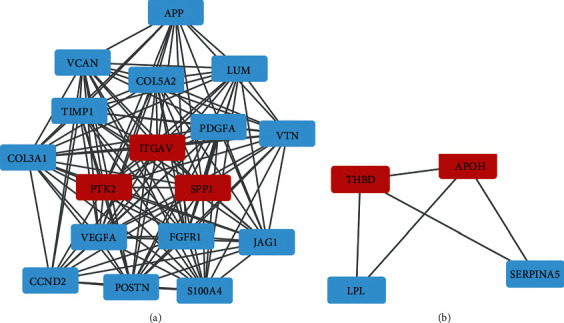
The (a) subnetwork 1 and (b) subnetwork 2 of protein-protein interaction network through MCODE of Cytoscape for N gene sets. The red represented the hub genes.

Using “Multiple proteins” module of STRING database (https://cn.string-db.org/), protein-protein interaction (PPI) networks were constructed for the two prognostic gene sets: T and N gene sets, respectively (Figures 8(a) and 8(b)). What is more, Cytoscape was exerted for further module analysis visualization. We obtained the close-related region clusters in PPI network by module MCODE (Molecular Complex Detection), and the results are plotted in Figures 9(a)–9(f) and 10(a) and 10(b) . For T gene set, the top three hub genes of subnetwork 1 were CXCL1, CXCL2, and CXCL8, subnetwork 2 were ALDOA, TALDO1, and LDHA, subnetwork 3 were CCL4, FCAR, and FCER1G, and the top two hub genes of subnetwork 4 were SAMSN1 and LILRB1, subnetwork 5 were SH3PXD2B and PPM1N, and subnetwork 6 were PKM and FKBP4. The hub genes of the clusters were mainly linked to immune reaction and metabolic process. As for N gene sets, the top three hub genes of subnetwork 1 were ITGAV, PTK2, and SPP1, and the top two hub genes of subnetwork 2 were THBD and APOH. These hub genes were mostly related to protein regulation.

## 4. Discussion

Cervical cancer has become the fourth most common cancer among women [24, 25]. Persistent infection with high-risk human papillomavirus (HPV) is a major risk factor for cervical cancer [26]. As a malignant tumor, the case fatality rate of cervical cancer increased, especially in young women, in China [27]. Therefore, it is important to discover the prognostic biomarkers in order to help diagnose and treat cervical cancer. On account of the less detectable of cervical cancer by Papanicolaou testing [28], there is a greater need to discover new subtypes of cervical cancer.

First of all, we used GSVA to calculate the enrichment scores of gene sets. Then, combined with NMF method, Cox regression model, and K-means clustering algorithm, we identified three novel subtypes of cervical cancer and validated in GEO cohort. Interestingly, patients in subtype 2 had significant poorer overall survival than patients in subtypes 1 and 3. Patients in subtypes 1 and 3 did not have significant difference in overall survival. Additionally, we found that there were significant variations between the three subtypes in histological type, T stage, M stage, and N stage.

Previous studies mainly concentrated on the subtypes of cervical cancer but ignored the role of adjacent nontumor tissue. And some of the studies also indicated that subtypes were important for the prognosis of cancers. The 5-year survival of neuroendocrine cervical cancer (NECC), a rare and aggressive subtype of cervical cancer [29], decreased as the stage increased, and for stage IVB, the five-year survival was 0% [30]. Pan et al. [31] and Cao et al. [32] revealed that the tumor histologic subtype was a prognostic factor of cervical cancer. Besides, a retrospective study discovered three main morphological subtypes [33]. Turashvili and Park [34] studied the subtypes of endocervical adenocarcinomas and helped clinical management. Li et al. [35] developed six subtypes based on DNA methylation sites. Therefore, developing new subtypes of cervical cancer is of great importance for the treatment and prognosis of cervical cancer.

Then, two prognosis-related gene sets, T gene set: T_GSE36888_UNTREATED_VS_IL2_TREATED_STAT5_AB_KNOCKIN_TCELL_2H_UP and N gene set: N_HALLMARK_ANGIOGENESIS, were identified through LASSO method. Moreover, in these two gene sets, high enrichment scores were all significantly related to worse overall survival time. N gene set contains genes upregulated during formation of blood vessels [15]. Therefore, genes from N gene set are related to angiogenesis. It is reported that angiogenesis helps the expansion of tumor tissues and is related to the progression of tumor [36–38]. Angiogenesis is associated with poor prognosis. So, inhibiting angiogenesis is reasonable to control tumor progression [39]. Genes from T gene set are upregulated in STAT5a-STAT5-b double knock-in (DKI) T cells, which hinder the formation of tetramers. These genes from T gene set are related to immune response. It is reported that the CD4+CD25+ T cells, NK cells, and CD8+ T cells of STAT5 DKI mice are deficient [40]. IL-2 growth factor can activate STAT5 [41]. Rani and Murphy showed that activated STAT5 plays a pivotal role in the proliferation of tumor cells and the suppression of antitumor immunity [42]. These two gene sets all showed poor survival and were all enriched in subtype 2, demonstrating that the bad prognosis of patients in subtype 2 may related to these two gene sets. This was consistent with the result of Figure 3(c).

Our study also found that the genes from T gene set were enriched in cytokine-cytokine receptor interaction, IL-17 signaling pathway, and viral protein interaction with cytokine and cytokine receptor pathways. These pathways are all related to metabolism and immune activities. Genes from T gene set also participate in response to lipopolysaccharide, response to molecule of bacterial origin, cytokine receptor binding, receptor ligand activity, signaling receptor activator, and neutrophil activation. For nontumor genes, they participate in glycosaminoglycan binding, heparin binding, and sulfur compound binding and are enriched in focal adhesion, PI3K-Akt signaling pathway, and proteoglycans in cancer.

The hub genes from T gene set included CXCL1, CXCL2, CXCL8, ALDOA, TALDO1, LDHA, CCL4, FCAR, FCER1G, SAMSN1, LILRB1, SH3PXD2B, PPM1N, PKM, and FKBP4. The hub genes of the clusters were mainly linked to immune reaction and metabolic process. It is reported that CXCL1, CXCL2, and CXCL8 are related to the tumor growth in cervical cancer [43, 44]. ALDOA may increase the possibility of progression of cervical cancer [45]. In addition, LDHA [46], CCL4 [47], and PKM [48] were reported to be associated with the development, proliferation, or progression of cervical cancer cells.

As for N gene sets, the hub genes included ITGAV, PTK2, SPP1, THBD, and APOH. These hub genes were mostly related to protein regulation. PTK2 [49] SPP1 [50] were also related to the progression of cervical cancer.

There were some limitations in our study. First of all, there were only 3 adjacent nontumor samples in TCGA cohort, and the others were tumor samples. Therefore, these results need to be validated after recruiting more normal people. Second, the two prognostic gene sets were not validated within clinical samples. Further work needs to be done to focus on investigating the clinical value of these gene sets. As a result, more researches should be done to verify these results.

## 5. Conclusion

In conclusion, our study discovered three novel subtypes of cervical cancer and identified two prognostic gene sets of cervical cancer. The hub genes of the two prognostic gene sets were also identified. Our study presented a theoretical foundation for other researchers to find better therapy strategies for cervical cancer patients. Based on these findings, we can develop more and more effective treatments for cervical cancer patients. Based on the gene-enriched pathways, we can develop specific drugs targeting the pathways.

## Figures and Tables

**Table 1:** The correlation between clinical characteristics and subtypes.

	Total (*n* = 304)	Subtype 1 (*n* = 71)	Subtype 2 (*n* = 110)	Subtype 3 (*n* = 123)	*p* value
Age (years) (%)					
≤65	269 (88.49)	64 (90.14)	99 (90)	106 (86.18)	0.5825
>65	35 (11.51)	7 (9.86)	11 (10)	17 (13.82)	
HPV_status					
Negative	9 (2.96)	1 (1.41)	2 (1.82)	6 (4.88)	0.5031
Positive	169 (55.59)	43 (60.56)	61 (55.45)	65 (52.85)	
Unknown	126 (41.45)	27 (38.03)	47 (42.73)	52 (42.28)	
Histological type (%)					
Nonsquamous	50 (16.45)	5 (7.04)	7 (6.36)	38 (30.89)	<0.001
Squamous	227 (74.67)	59 (83.1)	91 (82.73)	77 (62.6)	
Unknown	27 (8.88)	7 (9.86)	12 (10.91)	8 (6.5)	
Grade (%)					
G1	18 (5.92)	1 (1.41)	6 (5.45)	11 (8.94)	0.0667
G2	135 (44.41)	27 (38.03)	51 (46.36)	57 (46.34)	
G3	118 (38.82)	37 (52.11)	38 (34.55)	43 (34.96)	
G4	1 (0.33)	1 (1.41)	0 (0)	0 (0)	
Unknown	32 (10.53)	5 (7.04)	15 (13.64)	12 (9.76)	
TNM stage (%)					
Stage I	162 (53.29)	36 (50.7)	55 (50)	71 (57.72)	0.275
Stage II	69 (22.7)	20 (28.17)	21 (19.09)	28 (22.76)	
Stage III	45 (14.8)	10 (14.08)	22 (20)	13 (10.57)	
Stage IV	21 (6.91)	2 (2.82)	10 (9.09)	9 (7.32)	
Unknown	7 (2.3)	3 (4.23)	2 (1.82)	2 (1.63)	
T (%)					
T1	140 (46.05)	35 (49.3)	43 (39.09)	62 (50.41)	0.0376
T2	71 (23.36)	23 (32.39)	19 (17.27)	29 (23.58)	
T3	20 (6.58)	4 (5.63)	9 (8.18)	7 (5.69)	
T4	10 (3.29)	0 (0)	6 (5.45)	4 (3.25)	
Tis	1 (0.33)	0 (0)	1 (0.91)	0 (0)	
Unknown	62 (20.39)	9 (12.68)	32 (29.09)	21 (17.07)	
M (%)					
M0	116 (38.16)	37 (52.11)	32 (29.09)	47 (38.21)	0.0227
M1	10 (3.29)	1 (1.41)	3 (2.73)	6 (4.88)	
Unknown	178 (58.55)	33 (46.48)	75 (68.18)	70 (56.91)	
N (%)					
N0	133 (43.75)	35 (49.3)	40 (36.36)	58 (47.15)	0.0160
N1	60 (19.74)	20 (28.17)	23 (20.91)	17 (13.82)	
Unknown	111 (36.51)	16 (22.54)	47 (42.73)	48 (39.02)	

Note: TNM: primary tumor (T), regional lymph nodes (N), distant metastases (M).

**Table 2:** Risk coefficients of screened gene sets.

Gene sets	Coefficient
T_GSE36888_UNTREATED_VS_IL2_TREATED_STAT5_AB_KNOCKIN_TCELL_2H_UP	2.617
N_HALLMARK_ANGIOGENESIS	4.859875

## Data Availability

The transcriptome data from TCGA-CESC (HT-Sequence-FPKM) were downloaded from TCGA website (http://portal.gdc.cancer.gov). The clinical data of cervical cancer in TCGA database were downloaded from UCSC Xena (http://xena.ucsc.edu/). The cervical cancer datasets GSE44001 were downloaded from Gene Expression Omnibus (GEO) database (https://www.ncbi.nlm.nih.gov/geo/). The data of gene expression in TCGA cohort contained 3 adjacent nontumor samples and 306 tumor samples, and GEO cohort contained 300 tumor samples. The clinical data contained 308 samples in TCGA cohort after removing duplicates. 4922 gene sets were downloaded from MSigDB [15] for GSVA analysis.

## Abbreviations

GSVA: Gene set variation analysis
NMF: Nonnegative matrix factorization method
LASSO: Least absolute shrinkage and selection operator
GO: Gene Ontology
KEGG: Kyoto Encyclopedia of Genes and Genomes
STRING: Search Tool for the Retrieval of Interacting Genes
TCGA: The Cancer Genome Atlas
GEO: Gene Expression Omnibus
BP: Biological processes
CC: Cellular components
MF: Molecular functions
PPI: Protein-protein interaction
MCODE: Molecular Complex Detection.

## Supplementary Materials

Supplementary Materials See Figures S1 and Table S1 in the “Supplementary materials. docx” for comprehensive image analysis. Figure S1: identification of clinically relevant subtypes of cervical cancer in GEO database. (A) The optimal number of clusters (*k*). (B) The cluster plot of 3 subtypes. (C) Kaplan-Meier plot of the three clusters. (D) The correlation heatmap of 3 subtypes. (E) Silhouette plot of three subtypes. Table S1: the screened gene sets after univariable Cox regression analysis.
