# Supplementary material for: Identification of Specific Cervical Cancer Subtypes and Prognostic Gene Sets in Tumor and Nontumor Tissues Based on GSVA Analysis
Source: J Oncol. 2022 Oct 15;2022:6951885. doi: 10.1155/2022/6951885 (PMC9588340; doi:10.1155/2022/6951885)
Supplement: Supplementary Materials — See Figures S1 and Table S1 in the “Supplementary materials. docx” for comprehensive image analysis. Figure S1: identification of clinically relevant subtypes of cervical cancer in GEO database. (A) The optimal number of clusters (k). (B) The cluster plot of 3 subtypes. (C) Kaplan-Meier plot of the three clusters. (D) The correlation heatmap of 3 subtypes. (E) Silhouette plot of three subtypes. Table S1: the screened gene sets after univariable Cox regression analysis. [file 6951885.f1.pdf]

Supplementary materials

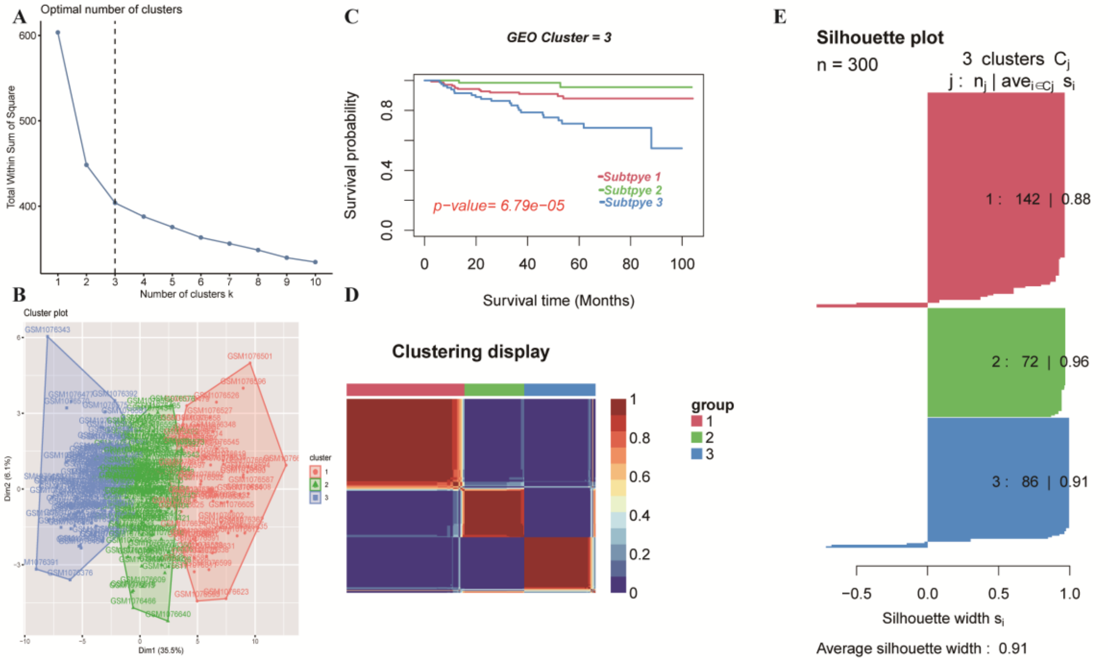

Figure S1. Identification of clinically relevant subtypes of cervical cancer in GEO database. (A) The optimal number of clusters (*K*). (B) The cluster plot of 3 subtypes. (C) Kaplan-Meier plot of the three clusters. (D) The correlation heatmap of 3 subtypes. (E) Silhouette plot of three subtypes.

Table S1. The screened gene sets after univariable cox regression analysis.

Gene sets and pathways	HR	ln(HR)	HR 95% CL_L	ln(HR 95% CL_L)	HR 95% CL_U	ln(HR 95% CL_U)	p-value
T_GSE36888_UNTREATED_VS_IL2_TREATED_STAT5_AB_KNOCKIN_TCELL_2H_UP	3297.754	8.100	8.683	2.161	1252518	14.041	0.0075
N_HALLMARK_ANGIOGENESIS	665.258	6.500	17.521	2.863	25258.59	10.137	0.00046
N_HALLMARK_EPITHELIAL_MESENCHYMAL_TRANSITION	43.790	3.779	2.516	0.923	762.245	6.636	0.0095
